# Potential association of LMNA-associated generalized lipodystrophy with juvenile dermatomyositis

**DOI:** 10.1186/s40842-018-0058-3

**Published:** 2018-03-27

**Authors:** Melis Sahinoz, Shafaq Khairi, Ashley Cuttitta, Graham F. Brady, Amit Rupani, Rasimcan Meral, Marwan K. Tayeh, Peedikayil Thomas, Meredith Riebschleger, Sandra Camelo-Piragua, Jeffrey W. Innis, M. Bishr Omary, Daniel E. Michele, Elif A. Oral

**Affiliations:** 1 Faculty of Medicine, Hacettepe University, Ankara, Turkey; 2 Metabolism Endocrinology and Diabetes Division, Department of Internal Medicine, University of Michigan and Brehm Center for Diabetes, 1000 Wall Street, Room 5313, Ann Arbor, MI MI48105 USA; 3 Department of Molecular and Integrative Physiology, University of Michigan, Ann Arbor, MI USA; 4 Division of Gastroenterology, Department of Internal Medicine, University of Michigan, Ann Arbor, MI USA; 5 Division of Genetics, Metabolism & Genomic Medicine, Department of Pediatrics and Communicable Diseases, University of Michigan, Ann Arbor, MI USA; 6 Division of Pediatric Rheumatology, Department of Pediatrics and Communicable Diseases, University of Michigan, Ann Arbor, MI USA; 7 Pathology Department, University of Michigan, Ann Arbor, MI USA; 8 Department of Human Genetics, University of Michigan, Ann Arbor, MI USA

**Keywords:** Laminopathies, Myositis, *LMNA*, Whole exome sequencing, P.T10I, Muscle biopsy

## Abstract

**Background:**

Juvenile dermatomyositis (JDM) is an auto-immune muscle disease which presents with skin manifestations and muscle weakness. At least 10% of the patients with JDM present with acquired lipodystrophy. Laminopathies are caused by mutations in the lamin genes and cover a wide spectrum of diseases including muscular dystrophies and lipodystrophy. The p.T10I *LMNA* variant is associated with a phenotype of generalized lipodystrophy that has also been called atypical progeroid syndrome.

**Case presentation:**

A previously healthy female presented with bilateral proximal lower extremity muscle weakness at age 4. She was diagnosed with JDM based on her clinical presentation, laboratory tests and magnetic resonance imaging (MRI). She had subcutaneous fat loss which started in her extremities and progressed to her whole body. At age 7, she had diabetes, hypertriglyceridemia, low leptin levels and low body fat on dual energy X-ray absorptiometry (DEXA) scan, and was diagnosed with acquired generalized lipodystrophy (AGL). Whole exome sequencing (WES) revealed a heterozygous c.29C > T; p.T10I missense pathogenic variant in *LMNA,* which encodes lamins A and C. Muscle biopsy confirmed JDM rather than muscular dystrophy, showing perifascicular atrophy and perivascular mononuclear cell infiltration. Immunofluroscence of skin fibroblasts confirmed nuclear atypia and fragmentation.

**Conclusions:**

This is a unique case with p.T10I *LMNA* variant displaying concurrent JDM and AGL. This co-occurrence raises the intriguing possibility that *LMNA*, and possibly p.T10I, may have a pathogenic role in not only the occurrence of generalized lipodystrophy, but also juvenile dermatomyositis. Careful phenotypic characterization of additional patients with laminopathies as well as individuals with JDM is warranted.

**Electronic supplementary material:**

The online version of this article (10.1186/s40842-018-0058-3) contains supplementary material, which is available to authorized users.

## Background

Juvenile dermatomyositis (JDM) is an autoimmune inflammatory disease, which typically affects the proximal skeletal muscles and presents with characteristic cutaneous manifestations in the first 18 years of life [1]. Among other multi-system manifestations, association with abnormal fat distribution and metabolic abnormalities has been recognized to fit the phenotype of lipodystrophy. The reported prevalence of lipodystrophy in patients with JDM is 8–40% In a large multi-center study where 353 patients with JDM were evaluated for having lipodystrophy, 28 JDM patients were found to have lipodystrophy, either generalized or partial [1].

Acquired generalized lipodystrophy (AGL) is characterized by progressive loss of subcutaneous adipose tissue, which starts before adolescence. The fat loss affects the whole body and results in low serum leptin levels [1, 2]. As a consequence, patients experience various metabolic complications such as hypertriglyceridemia, hyperphagia, severe insulin resistance leading to diabetes, and non-alcoholic steatohepatitis. These metabolic derangements often result in serious complications such as recurrent episodes of acute pancreatitis, premature cardiovascular disease, and hepatic cirrhosis [1, 3].

Mutations in the *LMNA* gene cause a wide spectrum of diseases called laminopathies which include muscular dystrophies such as limb girdle muscular dystrophy type 1 (LGMD); progeroid syndromes like Hutchinson Gilford progeria syndrome (HGPS); and familial partial lipodystrophy (FPLD) which is a lipodystrophy syndrome [4, 5]. *LMNA* p.T10I results from a missense mutation (c.29C > T) in exon 1 and leads to a mutant lamin A protein. A total of nine cases presenting with this mutation, including the case reported herein, have recently been described in a case series as part of a global collaboration [6] with 5 cases presenting with a phenotype suggestive of AGL [4, 5, 7] and initially deemed to have either atypical progeroid syndrome or AGL. However, no other case with this mutation was reported to have lipodystrophy and JDM concurrently and none have undergone a muscle biopsy to the best of our knowledge.

Here, we present a unique patient harboring the heterozygous c.29C > T (p.T10I) mutation in lamin A/C who presented originally with JDM followed by what was diagnosed as AGL. This case provides evidence that the spectrum of muscle abnormalities caused by laminopathies may be wider than previously recognized.

## Methods

**Fig. 1:**
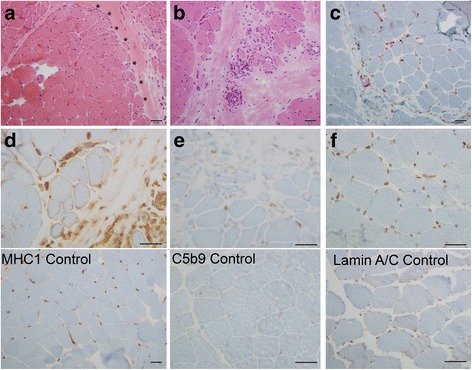
**a** Hematoxylin Eosin (HE) staining shows prominent perifascicular atrophy (asterisk). **b** HE staining highlights mononuclear infiltration around a blood vessel and infiltrating the endomysium. **c** CD163/CD4 immunostaining highlights several CD4 T-cells (red) in the perivascular and endomysial space with associated macrophages (brown) (**d**) MHC1 immunostaining shows abnormal sarcolemmal membrane overexpression in myocytes, note the normal control below should only highlight capillaries (**e**) C5b9 immunostaining highlights complement deposition in capillaries associated with affected fibers; note normal control does not show any capillary immunostain (**f**) Lamin A/C staining shows nuclear staining comparable to normal control. Scale bars: 50 μm

**Fig. 2:**
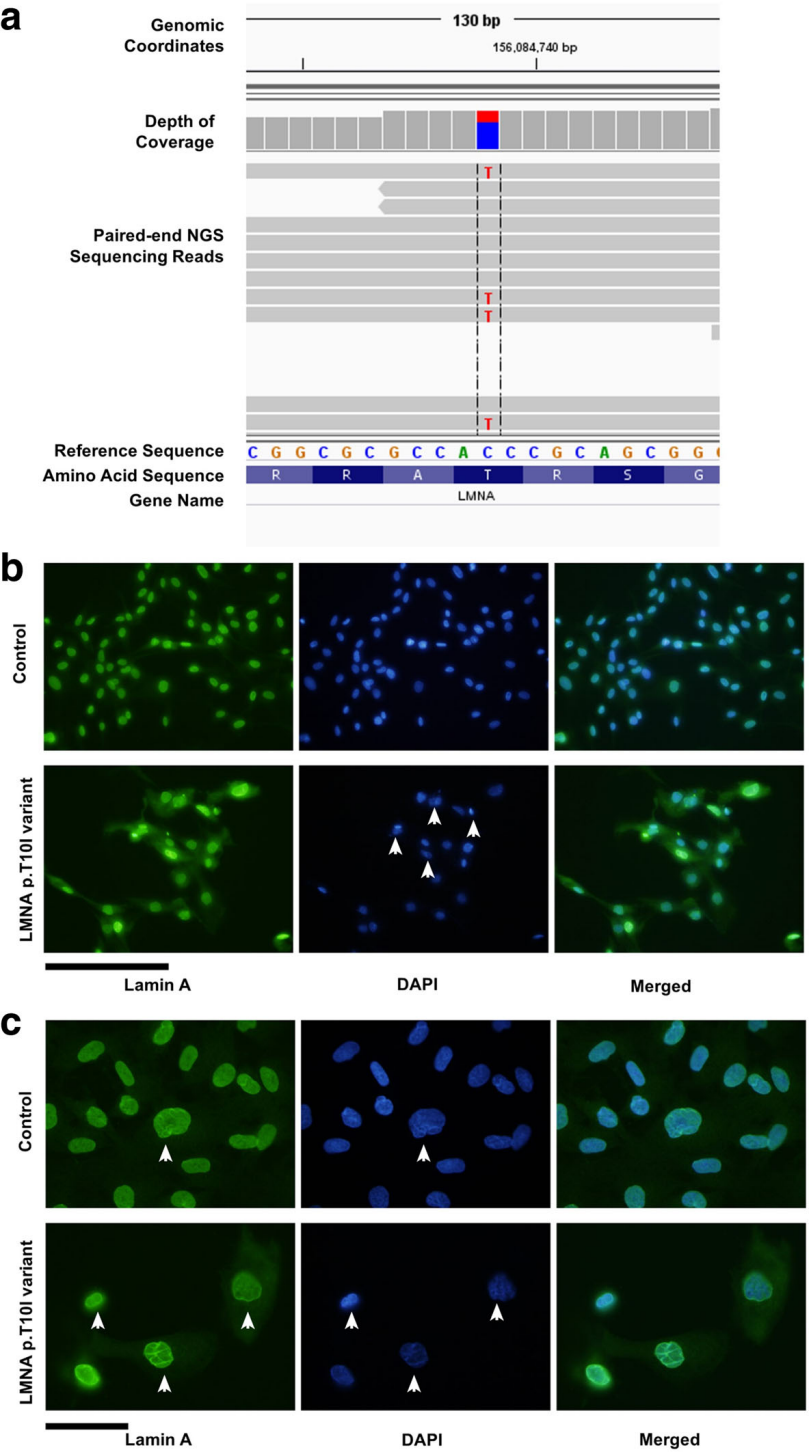
p.T10I *LMNA* mutation disrupts nuclear structure. **a** Integrated Genomics Viewer (IGV) screenshot of 130 bp paired-end whole exome sequencing reads, represented by horizontal gray bars, demonstrated a single nucleotide change (C to T) at genomic position of chromosome 1 g.156,084,738 (GRCh37/hg19) resulting in a missense alteration at codon 29 of LMNA replacing a threonine (T) with an isoleucine (I): NM_170707.3(LMNA):c.29C > T (p.T10I) (**b**) Skin fibroblast cell cultures of our patient with heterozygous p.T10I *LMNA* mutation and a Duchenne muscular dystrophy patient were analyzed by immunoflourescence using an antibody to lamin A. Cell cultures were stained with primary lamin A antibody, followed by 4′,6-diamidino-2 phenylindole (DAPI) nuclear staining and with the Alexa 488 goat anti-rabbit IgG. Scale bar: 200 μm. **c** Higher magnification images of the nuclear staining shown in (**b**); scale bar: 50 μm. Arrows in panel (**b**) indicate fragmented nuclei; arrows in panel (**c**) indicate examples of atypically shaped nuclei and/or nuclei with ruffles of lamin A staining

Our patient’s metabolic characteristics were previously reported in a paper describing metabolic response of lipodystrophy patients to metreleptin. Methods for metabolic characterization were also included in this report [2]. Informed assent and consent were obtained from the patient and her mother for genetic testing and fibroblast collection, as well as from the control patient with Duchenne muscular dystrophy under approval from the Institutional Review Board of the University of Michigan Medical School. Histopathological examination of fresh frozen muscle biopsy specimen (Fig. 1) was performed using standard Hematoxyin Eosin(HE) staining and immunostains for CD3/CD20, CD163/CD4, C5b9, MHC1, Lamin A/C, following standard procedures with specific antibodies (Additional file 1: Appendix 1). Whole exome sequencing (WES) (Fig. 2) and confirmation via Sanger sequencing were performed as previously described [8] (Additional file 1: Appendix 2). Skin fibroblast cultures obtained from the patient and a control Duchenne muscular dystrophy patient were analyzed via indirect immunofluorescence with the lamin A antibody [9].

## Case presentation

The patient is a 17 year-old African-American female, who had normal fat development until age 4 when she started losing weight and muscle strength. At age 6, she presented with bilateral proximal lower extremity weakness and had elevated levels of erythrocyte sedimentation rate (ESR), C-reactive protein (CRP), creatinine kinase (CK), lactate dehydrogenase (LDH), aspartate aminotransferase (AST) and aldolase. T2-weighted (magnetic resonance imaging).

MRI images of her lower extremities showed hyperintensity in her quadriceps muscles consistent with myositis. On the basis of her clinical presentation, laboratory results and MRI, she was diagnosed with JDM. Her presentation was reported previously [2]. On physical examination, she had generalized loss of subcutaneous fat including her face, a high-arched palate, micrognathia, a small mid-face and bilateral parotid enlargement. She also had progressive contractures in most upper extremity joints, calcinosis on her knuckles and mottled skin throughout her upper body. She had hepatosplenomegaly and non-alcoholic steatohepatitis. Her leptin level was < 0.7 ng/dL, and her DEXA scan showed 9.3% body fat. She was diagnosed with AGL, with associated diabetes and polycystic ovarian syndrome. In the next 7 years, she had severe elevations in triglycerides > 10,000 mg/dL and multiple episodes of acute pancreatitis and diabetic ketoacidosis. Echocardiogram performed at age 9 showed moderate aortic stenosis and atrial septal defect, ostium secundum type. She also had ovarian cysts and nephromegaly due to bilateral renal cysts. Despite being on methotrexate for JDM, her serum muscle enzyme levels fluctuated. For her lipodystrophy, she was treated with metreleptin [2].

Whole exome sequencing done at age 16 identified a heterozygous missense mutation (c.29C > T) in *LMNA* resulting in p.T10I substitution which is considered to be pathogenic (Fig. 2). This was not maternally inherited. The patient’s father had sudden cardiac death at age 49, so a blood sample was not available. He was reported to be thin, especially on the face and was diabetic in the last 2 years of his life. Further he had history of thyroid cancer as conveyed by patient and her mother. The patient’s WES data was interrogated for pathogenic variants in the genes that might be related to JDM, such as TNF, HLADRB1, IL1RN, IL1A, IL1B, ISG15, BLK and CCL21. No pathogenic variants were found in these genes. Of note, PLCL1 gene was not covered in the patient’s WES data, hence was not screened. A muscle biopsy was obtained from her right quadriceps muscle which confirmed the earlier diagnosis of JDM, including perifascicular atrophy, perivascular mononuclear cell infiltrates and upregulation of sarcolemmal MHC1 (Fig. 1). Immunofluorescence staining of skin fibroblasts from the patient and a control patient with Duchenne muscular dystrophy which is used as a non- LMNA related control both displayed positive nuclear staining for lamin A (Fig. 2). However, *LMNA* mutant nuclei exhibited greater nuclear atypia and fragmentation compared to the control fibroblasts from an unrelated myopathy without LMNA mutations. Our results in Fig. 2 are consistent with previous reports that scored atypical nuclei which reported that control fibroblasts have occaisonal atypical nuclei in ~ 19% of cells while the number of atypical nuclei in fibroblasts from patients with lamin A/C mutations were much more common (33–92%) [8]. It should be noted that it is very reasonable to use Duchenne muscular dystrophy (DMD) as a control, and although there is one report of a case study of a DMD patient with altered nuclear structure similar to LMNA related muscular dystrophy, further analysis showed this patient also carried a mutation in Nesprin-1, and patients with other mutations in dystrophin showed no significant differences in nuclear structure from non-disease human fibroblasts [10].

## Discussion

Here we report a patient with concurrent JDM and AGL with some atypical features. She was found to have a p.T10I mutation in the lamin A/C region encoding the N-terminal head; such mutations were previously associated with generalized lipodystrophy in patients classified as having atypical progeroid syndrome [4, 5, 7]. To our knowledge, she is the first with this mutation to present with both generalized lipodystrophy and biopsy proven JDM. In fact, the nine cases observed including our case were summarized in a new paper recently and none of the others presented with muscle disease [6]. Because different lamin A/C mutations cause various muscle disorders as well as lipodystrophy phenotypes [5], we propose that the presence of JDM and AGL might be linked to the p.T10I mutation in this patient.

The wide spectrum of diseases within the laminopathies is attributed to different mutations in *LMNA* leading to various modifications in the tertiary structure of the lamin A/C proteins. In addition to disruptions in the nuclear structure leading to fragility, some mutations are known to interfere with cell division, alter interactions with certain transcription factors, or modify gene expression (8). It was proposed earlier that the p.T10I mutation, which is located in the extreme amino terminus of the lamin A protein, might have a causal role in detachment of chromatin from the nuclear lamina as well as disruption of lamin polymerization [5, 8]. Four previous reports associate inflammatory myositis with *LMNA* variants, suggesting that the muscle disease of laminopathy is not restricted to muscular dystrophy and may be more complicated [11–14].

While muscle biopsies are now rarely performed in suspected cases of *LMNA* mutations causing muscular dystrophy, previous reports have shown that some *LMNA* muscular dystrophy patients have marked inflammatory cell infiltrates, although without noted upregulation of MHC [15]. More careful analysis of potential causes of the muscle pathology may reveal greater overlap between the lipodystrophy syndromes, myositis, and muscle diseases associated with lamin A/C mutations than previously suggested. Based on previous work, JDM appears to be a heterogeneous phenotype and to date, no clear molecular etiology has emerged although HLA associations and some genome wide association study (GWAS) loci have been reported [16]. Our case and a few other published reports [12–14] raise the possibility that nuclear lamins may be implicated in the development of inflammatory myopathies. We propose that deeper muscular phenotyping of laminopathies in patients who present with this genotype should be undertaken to understand if we are correct especially if abnormalities in muscle enzymes can be demonstrated. The selective genetic modifiers that result in different tissue manifestations despite having the same mutation remain to be defined.

## Conclusion

*LMNA* mutations are associated with an array of diseases called laminopathies which include muscular dystrophy and lipodystrophy, among other diseases. However, no certain causal relation between *LMNA* mutations and inflammatory muscle diseases has been established. We posit that both inflammatory muscle disease and generalized lipodystrophy in this patient are due to the lamin A/C T10I variant, although a multifactorial etiology cannot be excluded. This case broadens the spectrum of laminopathies; more importantly, it provides the first potential molecular link to the known association between JDM and lipodystrophy syndromes. Further studies probing the interaction between nuclear envelope proteins and the immunotranscriptome are needed to unlock the mysteries of this long recognized but unexplained association.

## Additional file

Additional file 1: Appendix 1. Antibodies used for immunohistochemistry. Histopathological examination using fresh frozen muscle biopsy specimen from the patient was performed using the immunostains for: MHC1, Lamin A/C, C5b9, CD163/CD4, CD3/CD20. The antibodies used and respective protocols are shown. Appendix 2 Sanger sequencing chromatogram completed by a CLIA certified laboratory. Data from Sanger sequencing was used to confirm the patient’s whole exome sequencing (WES) results. Similar to the WES data, Sanger sequencing chromatogram revealed a heterozygous c.29 C > T mutation in exon 1. In the figure, both the wild type and the patient’s data with this mutation are shown. (PDF 618 kb)

## Abbreviations

AGL: Acquired generalized lipodystrophy
AST: Aspartate aminotransferase
BLK: Blymphocyte kinase
c.29C > T: Replacement of a cytosine with a thymidine at codon 29
C5b9: Complement membrane attack complex
CCL21: Chemokine (C-C motif) ligand 21
CD: Cluster of differentiation 3
CK: Creatinine kinase
CRP: C-reactive protein
DEXA: Dual energy X-ray absorptiometry
ESR: Erythrocyte sedimentation rate
FPLD: Familial partial lipodystrophy
GWAS: Genome wide association study
HGPS: Hutchinson Gilford progeria syndrome
HLA DRB1: Human leukocyte antigen DRB1
IL1A: Interleukin 1 alpha
IL1B: Interleukin 1 beta
IL1RN: Interleukin 1 receptor antagonist
ISG15: Interferon stimulated gene 15
JDM: Juvenile dermatomyositis
LDH: Lactate dehydrogenase
LGMD: Limb girdle muscular dystrophy
MHC1: Major histocompatibility complex class I
MRI: Magnetic resonance imaging
p.T10I: Replacement of threonine with isoleucine at position 10
PLCL1: Phospholipase C Like 1
TNF: Tumor necrosis factor
WES: Whole exome sequencing
