# Supplementary material for: Potential association of LMNA-associated generalized lipodystrophy with juvenile dermatomyositis
Source: Clin Diabetes Endocrinol. 2018 Mar 27;4:6. doi: 10.1186/s40842-018-0058-3 (PMC5870259; doi:10.1186/s40842-018-0058-3)
Supplement: Supplementary file 1 — Appendix 1. Antibodies used for immunohistochemistry. Histopathological examination using fresh frozen muscle biopsy specimen from the patient was performed using the immunostains for: MHC1, Lamin A/C, C5b9, CD163/CD4, CD3/CD20. The antibodies used and respective protocols are shown. Appendix 2 Sanger sequencing chromatogram completed by a CLIA certified laboratory. Data from Sanger sequencing was used to confirm the patient’s whole exome sequencing (WES) results. Similar to the WES data, Sanger sequencing chromatogram revealed a heterozygous c.29 C > T mutation in exon 1. In the figure, both the wild type and the patient’s data with this mutation are shown. (PDF 618 kb) [file 40842_2018_58_MOESM1_ESM.pdf]

Appendix 1

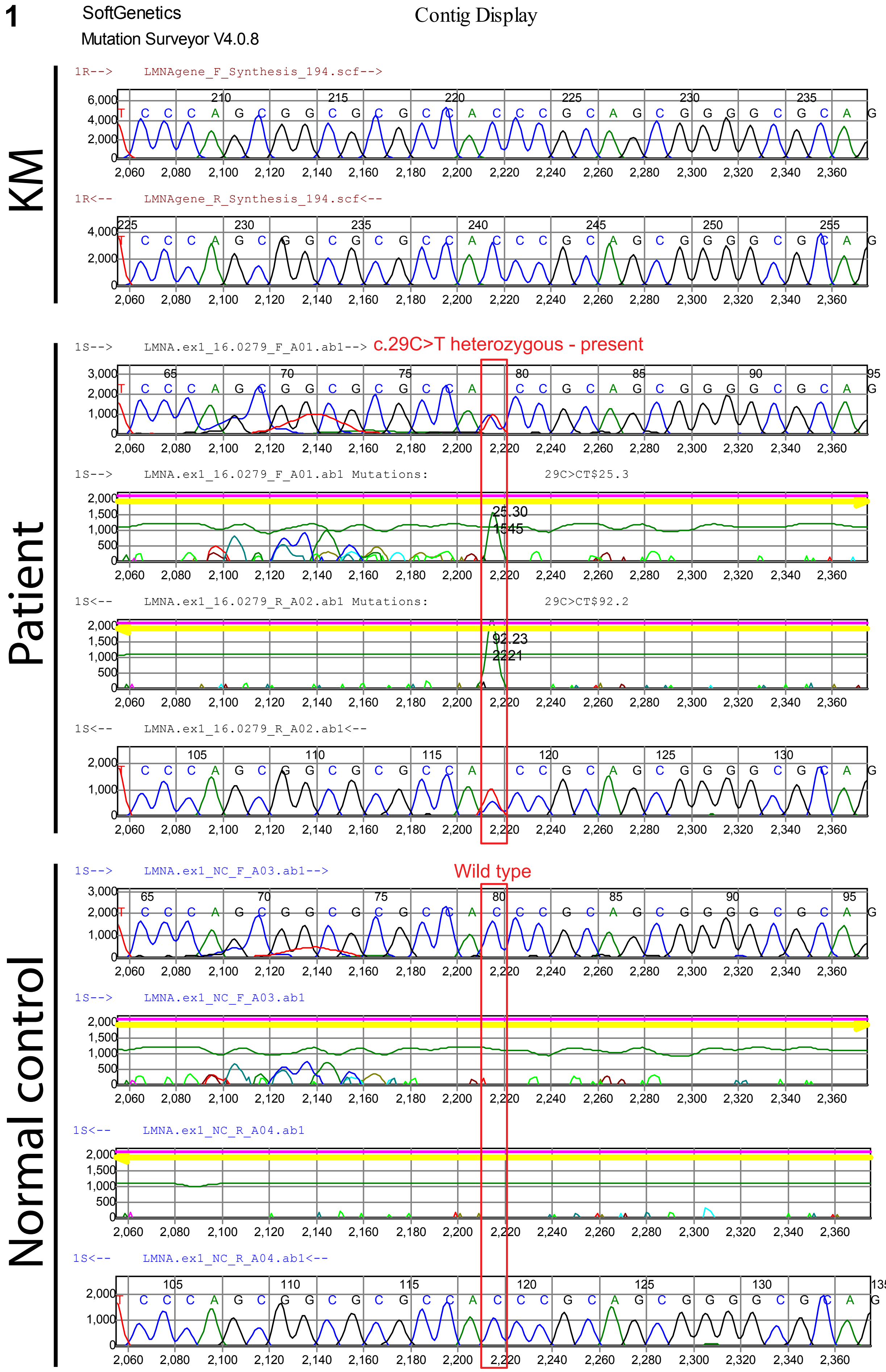

Appendix 1. Sanger sequencing chromatogram completed by a CLIA certified laboratory

Appendix 2

Antibody	Clone, source and dilution	Retrieval Protocol
MHC1	HLA-ABC (Clone W6/32) Dako cat# M0736 used at a dilution of 1:1000	I-View DAB Kit, 44 minutes primary antibody incubation (no heat)
LAMIN A/C	(Clone 131C3) Millipore cat# MAB3538 used at a dilution of 1:50	I-View DAB Kit, 44 minutes primary antibody incubation (no heat)
C5b9	(Clone aE11) Dako cat# M0777 used at a dilution of 1:50	UltraView DAB Kit, Ultra CC1 36 minutes, 32 minutes primary antibody incubation at 37°C
CD163	(Clone 10D6) Leica cat# NCL-CD163 used at a dilution of 1:100	UltraView DAB Kit, Ultra CC1 36 minutes, 32 minutes primary antibody incubation at 37°C
CD3	(Clone 2GV6) Ventana cat# 790-4341 (pre-dilute)	UltraView DAB Kit, Ultra CC1 36 minutes, 28 minutes primary antibody incubation at 36°C
CD20	(Clone L26) Ventana cat# 760-2531 (pre-dilute)	UltraView DAB Kit, Ultra CC1 20 minutes, 32 minute primary antibody incubation at 36°C, UltraWash

Appendix 2. Antibodies used for immunohistochemistry
